# Bactericidal and Cytotoxic Activities of Polyphenol Extracts from *Solanum tuberosum* spp. *tuberosum* and spp. *andigena* Cultivars on *Escherichia coli* and Human Neuroblastoma SH-SY5Y Cells *In Vitro*

**DOI:** 10.1155/2018/8073679

**Published:** 2018-03-22

**Authors:** María Ximena Silveyra, María Luciana Lanteri, Rocío Belén Damiano, Adriana Balbina Andreu

**Affiliations:** ^1^Instituto de Investigaciones Biológicas, Universidad Nacional de Mar del Plata-CONICET, CC 1245, 7600 Mar del Plata, Argentina; ^2^Instituto Nacional de Epidemiología “Dr. Juan H. Jara”, 7600 Mar del Plata, Argentina

## Abstract

Potatoes (*Solanum tuberosum* L.) are a good source of dietary antioxidant polyphenols. This study investigated the potential antioxidant, bactericidal, and cytotoxic activities *in vitro* of the phenolic compounds present in tubers of one *S. tuberosum* spp. *tuberosum* (Summerside), and three *S. tuberosum* spp. *andigena* (landraces Moradita, Waicha, and Santa María) cultivars. Both the content of phenolic acids, chlorogenic acids (CGAs) being the most abundant, and the antioxidant activity were higher in extracts from skin than from flesh. Extracts from Moradita flesh and Summerside skin showed bactericidal activity against *Escherichia coli* ATCC 25922 but failed to inhibit pathogenic *E. coli* O157. Both extracts lack pigmentation but do contain 5-CGA, caffeic, and ferulic acids. Positive control with gentamicin and commercial 5-CGA resulted in a complete inhibition of bacterial growth. In addition, all potato extracts and commercial 5-CGA diminished dose-dependently human neuroblastoma SH-SY5Y cell viability. Skin extracts were more potent than flesh extracts. Among flesh extracts, Moradita was the most potent. Together, our results suggest that Moradita flesh could provide a desirable source of important health-promoting substances. Findings indicate that the biological activity of potato extracts is a combination of various bioactive compounds and contribute to the revalorization of potato as a functional food.

## 1. Introduction

Potato (*Solanum tuberosum* L.) is currently one of the most important food crops worldwide. Andean potatoes (*S. tuberosum* L. ssp. *andigena*) show a wide variability in tuber shape, and skin and/or flesh color. They contain antioxidant phenolic compounds like phenolic acids and anthocyanins, the latter mostly absent in *S. tuberosum* L. ssp. *tuberosum* varieties. Chlorogenic acids (CGAs) are esters formed between caffeic acids (CAs) and quinic acids and represent the major phenolic acids in tubers followed by CAs [1–3].

Phenolic compounds are synthesized through the plant phenylpropanoid biosynthetic pathway, part of the secondary metabolism. They have a wide range of functions, such as providing pigmentation to flowers, fruits, seeds, and tubers; and they are involved in protection against both ultraviolet light and pathogenic microorganisms. Furthermore, several health-promoting and therapeutic effects of phenolic compounds have been reported in humans. Many of these effects result from the powerful free radical scavenging properties of these compounds [4]. For instance, chemical-based assays have shown the capacity of CGAs to scavenge 2, 2-diphenyl-1-picrylhydrazyl (DPPH) radicals, superoxide anions, hydroxyl radicals, and peroxylnitrite [5]. The presence of phytonutrients (compounds in plant-based foods that play a potentially beneficial role in the prevention and treatment of disease) makes potato tubers a promising functional food, and diets rich in potatoes likely have beneficial health effects [1].

The antimicrobial activity of phenolic compounds from various sources, among which potato tubers, has been studied. Different compounds exert different activities against an array of microorganisms. Particularly, their effects on both beneficial and pathogenic human intestinal bacteria have been extensively investigated [6–10]. Phenolics exhibit a broad spectrum of activities resulting from their radical scavenging action [11]. They can alter enzyme activities through reactions with sulfhydryl groups or through nonspecific interactions, alter the structure and function of the cytoplasmic membrane, and disrupt the proton motive force, electron flow, and active transport [7, 12]. Besides having beneficial effects on human health, these properties make that certain phenolic compounds act as natural antimicrobials in food preservation and, hence, are valuable for the industry.

Studies in several laboratories have reported that phenolic acids and anthocyanins are implicated in suppression of cancer cell proliferation *in vitro*, with little or no effect on the growth of normal cells. Phenolic compounds are therefore considered natural products beneficial to human health [9, 10, 13–15]. Extracts from fruits and vegetables including potatoes have been shown to exhibit antiproliferative activity towards multiple cancer cells [9, 10, 16–21].

We have recently studied the biosynthesis of phenolic compounds through a comparative analysis of skin and flesh tissues in commercially bred [22] and Andean [23] potatoes. A number of studies have investigated the biological activities of potato polyphenols [3, 4, 24, 25]. Nevertheless, their effects as antimicrobials against *Escherichia coli* and anticancer agents against human neuroblastoma cells have not been reported yet. The majority of the evidence to date concerns 5-CGA, the primary isomer of CGA, with a lesser amount of information available for minor CGA isomers and other phenolics. The objectives of this work were to characterize and measure the concentrations of phenolic acids and anthocyanidins in four potato cultivars and to evaluate their antioxidant, antibacterial, and antiproliferative activities *in vitro*. Our results indicate that potato tuber polyphenols are potential antimicrobial and anticancer agents.

## 2. Materials and Methods

### 2.1. Plant Material

**Figure 1:**
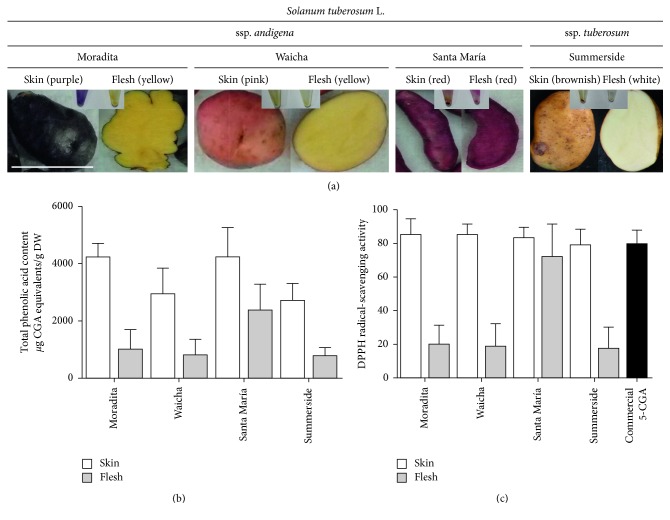
(a) Tubers from the four potato cultivars. Shown are entire tubers as well as cross sections and dry polyphenol extracts. Scale bar = 10 cm. (b) Total phenolic acids content and (c) DPPH radical scavenging activity, in skin (white bars) and flesh (grey bars) extracts. Positive control with commercial 5-CGA (0.5 *μ*g) was added (black bars). Values are presented as the mean ± SD of four independent experiments with at least three technical replicates each.

Moradita, Waicha, Santa María, and Summerside potato cultivars were selected based on previous work from our laboratory [22, 23]. The Moradita cultivar has purple skin and yellow flesh. Waicha has also yellow flesh but pink skin. The Santa María cultivar is intensely red in both skin and flesh. Summerside has a brownish skin and white flesh (Figure 1(a)).

Moradita, Waicha, and Santa María cultivars were grown in a field located in Yavi Department (22° 6′ 4″ S, 65° 35′ 44″ O, 3377 MAMSL), Jujuy, Argentina, during the 2012/2013 campaign. Summerside cultivar was grown in an experimental field located in Balcarce (37° 49′ 9.65″ S, 58° 13′ 11″ W, 130 MAMSL), Buenos Aires, Argentina, during the 2012/2013 campaign of McCain Argentina S.A. All cultivars were planted in random plots and harvested at the end of their respective cycles. For each cultivar, skin and flesh from freshly harvested tubers were pooled to generate a representative sample. The material was immediately frozen in liquid nitrogen and stored at −80°C until analysis. Frozen potato pieces were freeze-dried and finely powdered.

### 2.2. Phenolic Compound Extraction

Lyophilized tissue from potato tuber skin or flesh was incubated overnight with 100% methanol (0.2 g dry weight (DW)/4 mL) at 4°C in darkness with constant agitation. After centrifugation at 6000 g for 20 min at 4°C, extracts were completely evaporated in a rotational vacuum concentrator (RVC 2-18 CD plus, Martin Christ) and dissolved in 1 mL 30% methanol (v/v).

### 2.3. Total Phenolic Content Determination

Total phenolic content was determined according to a modified Folin-Ciocalteu method [26]. Briefly, 4 *μ*L of extract was diluted to 20 *μ*L with 30% methanol (v/v) and loaded on a 96-well plate. Each sample was mixed with 40 *μ*L of 10% Folin-Ciocalteu reagent (v/v) and incubated at room temperature in darkness for 5 min. Then, 160 *μ*L of 700 mM Na_2_CO_3_ was added and allowed to stand for color development for 10 min. Finally, absorbance at 725 nm was measured on a microplate spectrophotometer (Epoch BioTek), and contents were determined using a calibration curve ranging from 0.5 to 4 *μ*g of commercial 5-CGA (Sigma-Aldrich). Total phenolic acid content was expressed as *μ*g CGA equivalents/g DW. Blank samples consisted of 30% methanol (v/v). Contents were determined in triplicate for four independent extractions per sample.

### 2.4. DPPH Radical Scavenging Activity

Total hydrophilic antioxidant activity was evaluated using the DPPH radical scavenging assay described by Chan et al. [27] with slight modifications. Briefly, 5 *μ*L of extract was diluted to 50 *μ*l with 30% methanol (v/v) and loaded on a 96-well plate. Then, 150 *μ*L of freshly prepared 0.272 mM DPPH in 100% methanol was added and incubated at room temperature in darkness for 30 min. Finally, absorbance at 517 nm was measured on a microplate spectrophotometer (Epoch BioTek). Blank samples consisted of 30% methanol (v/v), and commercial 5-CGA (0.5 *μ*g) was used as a positive control. The scavenging activity was expressed as % of the control without extract. Activities were determined in triplicate for four independent extractions per sample.

### 2.5. Phenolic Acid and Anthocyanidin Analysis by HPLC-DAD

Quantification of phenolic compounds was carried out using a Shimadzu LC-Solution system equipped with a diode-array detector (DAD). A flow rate of 1 mL/min was used and 20 *μ*L samples, previously filtered through a 0.45 *μ*m PVDF syringe filter (Millipore), were injected onto a C-18 Phenomenex Luna column (250 × 4.6 mm i.d.; 5 *μ*m particle size). Identification was carried out by comparing retention times and spectra of authentic commercial standards. Monitoring was set at 320 nm (for CGA isomers and phenolic acids) and at 510 nm (for anthocyanidins). The external standard method of calibration and peak areas were used for quantification. Values were expressed as *μ*g/g DW from at least three independent extractions.

For CGA isomer analysis, the mobile phases were (A) acetonitrile and (B) HPLC-grade water acidified with HCl, pH 2.3. The solvent program was isocratic 90% B until 19 min, return to 0% B between 19 and 20 min and maintained until 25 min. Stock solutions of the reference standards were prepared at 400 ppm. Mixed calibration solutions were prepared ranging from 10 to 100 ppm for 5-CGA, cryptochlorogenic acid (4-CGA), and neochlorogenic acid (3-CGA) (Sigma-Aldrich).

For phenolic acid analysis, the mobile phases were (A) acetonitrile and (B) HPLC-grade water acidified with HCl (pH 2.3). The solvent program was 0 to 20 min gradient from 80 to 0% B, 20 to 25 min return to 80% B, and linear at 80% B until 30 min. Stock solutions of the reference standards were prepared at 400 ppm. Mixed calibration solutions were prepared ranging from 2 to 70 ppm for 5-CGA and CA and from 0.4 to 40 ppm for ferulic acid (FA) and coumaric acid (Sigma-Aldrich).

Anthocyanidins present in the samples were determined after acid hydrolysis, according to the procedure proposed by Durst and Wrolstad [28]. The mobile phases were (A) acetonitrile and (B) 4% H_3_PO_4_ in water. The solvent program was 0 to 25 min gradient from 85 to 75% B, 25 to 30 min gradient from 75 to 73% B, 30 to 30.5 min return to 85% B, and linear at 85% B until 33 min. Stock solutions of the reference standards were prepared at 200 ppm. Mixed calibration solutions were prepared ranging from 0.5 to 15 ppm for each individual anthocyanidin (delphinidin, cyanidin, petunidin, pelargonidin, peonidin, and malvidin) purchased in Extrasynthese.

### 2.6. Bactericidal Activity

Two *E. coli* strains were used for experiments. *E. coli* ATCC 25922 is a recommended reference strain for antibiotic susceptibility testing [29]. *E. coli* O157 is a pathogenic strain that causes food intoxication by verotoxin production [30]. Both strains were grown on tryptic soy agar plates and suspended in Mueller Hinton broth (DIFCO). The inoculum suspensions were adjusted to obtain a 0.5 McFarland standard turbidity. Extracts were filtered with a sterile membrane Millex-LG 0.22 *µ*m (Millipore) in laminar flow. Different dilutions of the sterilized extracts expressed as concentrations in *μ*g CGA equivalents/mL (determined by Folin-Ciocalteu reagent) were added to Mueller Hinton broth medium. The bacterial suspensions were aerobically incubated for 24 h at 37°C. Cultures containing sterile medium, extraction solvent (30% methanol v/v), gentamicin (Sigma-Aldrich), or commercial 5-CGA were used as controls. The determination of minimum bactericidal concentration (MBC) was carried out transferring aliquots of bacterial suspensions from the test tubes without turbidity to blood agar plates. Bacteria were subsequently dispersed, and colony counts were recorded. MBC values were calculated as the lowest concentration that results in 99.9% death of the bacteria being tested. The experiments were performed at least twice.

### 2.7. Cytotoxic Activity

Human neuroblastoma SH-SY5Y cells were maintained in DMEM (Sigma-Aldrich) supplemented with 10% fetal bovine serum (v/v) (Internegocios SA), penicillin (100 U/mL), streptomycin (100 *μ*g/mL), and amphotericin B (0.25 *μ*g/mL) at 37°C in humidified atmosphere containing 95% air and 5% CO_2_. For the experiments, cells were seeded in 96-well plates at 5 × 10^4^ cells/well for 48 h and subsequently incubated in the presence of different dilutions of the potato extracts expressed as concentrations in *μ*g CGA equivalents/mL (determined by Folin-Ciocalteu reagent) or of commercial 5-CGA. The final concentration of methanol in each well did not exceed 3% (v/v). The cytotoxic activity of the extracts was determined by the MTT assay [31]. After 24 h treatment, MTT reagent (Sigma-Aldrich) was added in the amount of 1 mg/mL and incubated at 37°C for 4 h. Formazan crystals were dissolved in 100% DMSO and measured at 570 nm in a microplate spectrophotometer (Epoch BioTek). The % of cellular viability was obtained normalizing to the values in absence of treatments. The extracts were tested in triplicate, and the experiment was performed thrice.

### 2.8. Statistical Analysis

Pearson product moment correlation coefficients (*r* values) were calculated with GraphPad and a significance level (alpha) of 0.05, using the means of total phenolic content and antioxidant activities.

## 3. Results

### 3.1. Total Phenolics and Antioxidant Activity of Potato Extracts

Total phenolic acid content was determined by the Folin-Ciocalteu reaction. Moradita and Santa María have similar and considerably higher levels of phenolic acids in skin, while Santa María cultivar has by far the highest levels of phenolic acids in flesh (Figure 1(b)).

Total antioxidant activity was evaluated using the DPPH radical scavenging assay, and it was higher in skin than in flesh for the Moradita, Waicha, and Summerside cultivars. In contrast, Santa María cultivar showed similar activities in skin and flesh extracts (Figure 1(c)). A positive and significant correlation was found between total phenolic acid contents and antioxidant activity levels in potato extracts (*r* = 0.92, *P* value for a two-tailed test = 0.0010).

### 3.2. Phenolic Acid and Anthocyanidin Composition by HPLC-DAD

The levels of six individual phenolic acids (5-CGA, 4-CGA, 3-CGA, CA, FA, and coumaric acid) and of the six individual anthocyanidins that occur in nature (delphinidin, cyanidin, petunidin, pelargonidin, peonidin, and malvidin) were quantified by HPLC-DAD in extracts of skin and flesh of the four potato cultivars (Table 1). Major peaks were quantified, as shown in representative HPLC-DAD chromatograms (Supplementary Figure S1), while some minor compounds remain to be identified.

The levels of all the phenolic acids were higher in skin than in flesh. For instance, 5-CGA levels were up to 15.4 times higher in Moradita skin, as indicated by the skin to flesh ratio (*R* value, Table 1).

HPLC-DAD analysis revealed that 5-CGA was the major phenolic acid (≥55.20%) in both skin and flesh of Moradita, Santa María, and Summerside cultivars (Table 1). Surprisingly, 4-CGA was the most abundant isomer in Waicha extracts (37.3% in skin, 50.6% in flesh), followed by 3-CGA and 5-CGA. Interestingly, 5-CGA was the only isomer detected in Moradita flesh. CA and FA were minor components representing up to 6.5% and 4.5%, respectively (Table 1), whereas coumaric acid could not be detected by HPLC-DAD.

The phenolic acid profiles in skin extracts of Moradita, Santa María, and Summerside are comparable but contrasted to that found in Waicha. Altogether, a high qualitative and quantitative variation in phenolic acid composition among flesh extracts was observed (Table 1).

Anthocyanidins accounted for up to 38.7% of total compounds presented in Table 1. Santa María showed the major abundance of total anthocyanidins in both skin and flesh, followed by Moradita and Waicha. None of the six anthocyanidins were detected in nonpigmented fleshes of Moradita and Waicha as well as in Summerside tubers (Table 1).

The purple skin of Moradita has predominantly petunidin (91.2%) followed by peonidin (4.4%), malvidin (4.1%), and trace amounts of cyanidin. As expected by their color, Waicha skin and Santa María skin and flesh had similar composition of anthocyanidins. These cultivars contain predominantly pelargonidin (up to 84.7%) followed by peonidin (up to 19.8%), malvidin (up to 5.2%), and trace amounts of cyanidin (Table 1). Delphinidin could not be detected by HPLC-DAD.

### 3.3. Inhibition of E. coli Growth by Potato Extracts

The potential growth inhibitory effect of the potato extracts was investigated using an *in vitro* assay against two *E. coli* strains, nonpathogenic ATCC 25922, and pathogenic O157.  Table 2 summarizes the values of minimum bactericidal concentration (MBC) obtained for both strains treated for 24 h. As expected, the negative control with extraction solvent (30% methanol v/v) did not affect bacterial growth. Sensitivity of the compounds was found to differ significantly among the tested organisms. In *E. coli* ATCC 25922, positive control with 1 *µ*g/mL gentamicin resulted in a total bactericidal effect, whereas MBC for commercial 5-CGA was 512 *µ*g/mL (Table 2). Pathogenic bacterium was more resistant to both gentamicin (MBC 2 *µ*g/mL) and 5-CGA (MBC 2048 *µ*g/mL). Then, extracts from Moradita flesh and from Summerside skin showed a total bactericidal activity against *E. coli* ATCC 25922 at 1024 *µ*g CGA equivalents/mL. The other extracts did not affect *E. coli* growth. Notably, *E. coli* O157 was completely resistant to all the potato extracts studied until a dose of 2048 *µ*g CGA equivalents/mL (Table 2). Similar results were obtained at 32 h of treatment (data not shown).

### 3.4. Potato Extracts Induce a Cytotoxic Effect in Human Neuroblastoma SH-SY5Y Cells

**Figure 2:**
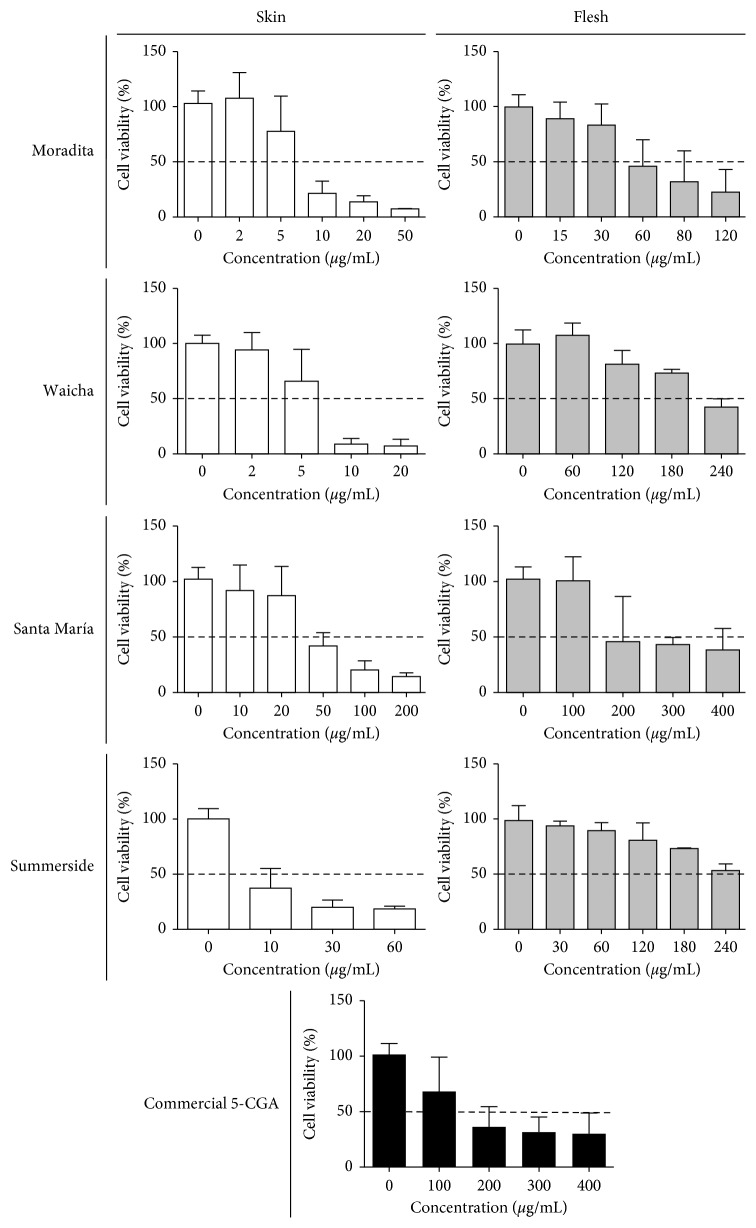
Cytotoxic activity of tuber extracts from potato cultivars in human neuroblastoma SH-SY5Y cells. Different concentrations (*μ*g CGA equivalents/mL) of skin (white bars) or flesh (grey bars) extracts from Moradita, Waicha, Santa María, and Summerside were studied. Positive control with commercial 5-CGA was added (black bars). Values are presented as the mean ± SD of at least three independent experiments with three technical replicates each.

The potential growth effect of the potato extracts was investigated using the MTT assay.  Figure 2 shows the effect of different concentrations of the extracts and commercial 5-CGA on the viability of neuroblastoma SH-SY5Y cells *in vitro*. As shown, all tested extracts caused dose-dependent inhibition of proliferation. Effective doses of skin extracts were approximately one order of magnitude lower than those observed for flesh extracts (Figure 2). When comparing skin extracts, the major cytotoxic effect was obtained with extract from Moradita and Waicha cultivars, followed by Summerside and finally Santa María. Among flesh extracts, Moradita was the most potent (50% viability at around 60 *µ*g CGA equivalents/mL), and the other three extracts all show 50% viability at around 200 *µ*g CGA equivalents/mL. Commercial 5-CGA had cytotoxic activity with effective concentrations comparable to flesh extracts (Figure 2).

## 4. Discussion

In the present study, extracts from tuber skin or flesh of four potato cultivars were analyzed by HPLC-DAD. It has been shown that Folin-Ciocalteu overestimates phenolic levels in samples [32], and the sum of the levels of the individual compounds determined by HPLC-DAD was indeed lower than the outcome of the Folin-Ciocalteu assay. However, we used the Folin-Ciocalteu data in order to be able to apply equivalent doses. In addition, the HPLC-DAD analyses depend on standards and, hence, will not be able to capture the complete spectrum of compounds. Both the concentration and composition of phenolic acids and anthocyanidins found in the extracts are in agreement with previous reports for pigmented [1, 20, 23, 33] and nonpigmented tubers [22, 34, 35]. Pigmented cultivars contained greater levels of CGAs than white/yellow-fleshed cultivars. CGAs were evidently the predominant phenolic acids with different concentrations and isomeric mixtures in skin and flesh. In general, 5-CGA comprised the majority of CGA isomers followed by 4-CGA and finally 3-CGA, as previously reported [2, 33, 36]. However, two cultivars showed unusual composition. In Waicha, 5-CGA was the least abundant isomer, while in Moradita flesh, it was the only isomer detected. According to Friedman [37] and Payyavula et al. [36], CGA isomer composition depends on environmental conditions such as light and temperature.

Our present results show that different potato extracts exert different biological activities. In summary, we demonstrated that extracts from Moradita flesh and Summerside skin have bactericidal activity against *E. coli* ATCC 25922. Both extracts lack anthocyanins but contain 5-CGA, caffeic, and ferulic acids. These findings indicate that the anthocyanins present in colored potato extracts are not implicated in the observed bactericidal activity. This is in agreement with two studies about the antimicrobial activity of purple potato (Vitelotte cultivar). Bactericidal effects from extracts against two Gram positive-species were found, using a total of 18 bacterial strains [9]. In concordance, Vitelotte extracts did not exhibit activity against entherotoxigenic *E. coli* DSM 8579 [10]. Nevertheless, microbial growth was inhibited in extracts after simulated gastrointestinal digestion [10]. On the contrary, reports on other plant species have shown that anthocyanins may be implicated in the antibacterial activity against *E. coli*. Red cabbage extract, a rich source of anthocyanins, decreased growth of both *E. coli* ATCC 25922 and *E. coli* O157:H7 strains (MBC 400 mg/mL) [38]. In a recent study, black mulberries had stronger antibacterial activity against *E. coli* than nonblack mulberries, which correlated with higher content of anthocyanins and flavonols [39]. Similar results were obtained with phenolic extracts from the seed coats of different colored soybean varieties against *E. coli* O157:H7 [40].

It is clear that the antibacterial activity may be attributed to the nature, level, and interactions between metabolites present in the extracts. For instance, our novel data related to the presence and abundance of the CGA isomers in the different potato cultivars may account for differences in bactericidal activity. Although all potato extracts contained 5-CGA, only two of them were bactericidal at a higher concentration (MBC 1024 *µ*g CGA equivalents/mL) than pure commercial 5-CGA (MBC 512 *µ*g/mL). For instance, Santa María flesh extract at 1024 *µ*g CGA equivalents/mL contains 500 *µ*g/mL of 5-CGA, which is similar to the concentration used with pure commercial 5-CGA. Therefore, we propose that antagonistic interactions between 5-CGA and other compound(s) present in the extracts could probably occur. Inhibitory doses for commercial 5-CGA are consistent with those found in the literature for an array of Gram-positive and Gram-negative bacteria [41, 42].

The presented data suggest that different strains of the same bacterial species have different sensitivities towards phenolics, since no bactericidal effects of the potato extracts were found against pathogenic *E. coli* O157, while commercial 5-CGA suppressed bacterial growth at a similar concentration (MBC 2048 *µ*g/mL) as was previously reported [43].

Previously, potato extracts were shown to induce a dose-dependent reduction in the cell viability of for instance breast, colon, prostate, and liver cancer lines [9, 10, 18–21]. Our observations show that potato extracts are also potent inducers of cell death in human neuroblastoma SH-SY5Y cells, forming promising agents in cancer therapy. Inhibition of cancer development by phenolic compounds could be due to their ability to scavenge free radicals, electrophiles, and toxic metals; to inhibit enzymes that activate precarcinogens to carcinogens; and to induce carcinogen-detoxifying enzymes [44].

Moradita and Santa María skin extracts showed comparable level and composition of phenolics, as determined by Folin-Ciocalteu reagent and HPLC-DAD. However, Santa María extracts were less cytotoxic against SH-SY5Y cells. This could be explained by the different concentration and composition of anthocyanins present in these extracts. Growth inhibition studies clearly showed that skin extracts from Moradita, Waicha, and Summerside, with dissimilar content and composition of phenolic acids and anthocyanins, were highly active with comparable antiproliferative activity. Therefore, the effects reported here were dependent on the genotype. This is consistent with previous data obtained with different potato extracts in human prostate cancer cells [18].

Usually, extracts with higher total phenolic content show greater antioxidant and antiproliferative activities [21]. In our experiments, potato skin extracts displayed higher cytotoxic effects on neuroblastoma SH-SY5Y cells than flesh extracts. Interestingly, Moradita flesh extract exerted a major effect compared to the other flesh extracts, being similar in both phenolics and antioxidant levels to Waicha and Summerside, and less than red-fleshed Santa María. Moradita flesh composition is unique since it has only 5-CGA and lacks 4-CGA and 3-CGA. However, *in vitro* antioxidant and antiproliferative activities of the three CGA isomers have been reported to be similar [45]. Together, our results suggest that Moradita flesh could provide a desirable source for important health-promoting substances. Since it has also bactericidal properties, Moradita flesh is a promising candidate in future research concerning active compounds.

Since commercial 5-CGA showed less antiproliferative effect than most of the extracts, we suggest that the activity of potato is a result of various bioactive compounds working collaboratively. The compounds might even exert their effect synergistically, as previously shown [19, 46]. Fractionation and/or isolation of individual compounds from tuber extracts will be carried out in order to identify the components responsible for the reported effects.

Altogether, potatoes represent promising sources of natural products with an added value for the food industry and human health.

## 5. Conclusions

In summary, both phenolic acid contents and antioxidant activities were higher in tuber skin than in flesh, CGAs forming the major phenolic acid in potato tuber. Potato extracts from Moradita flesh and from Summerside skin had bactericidal activity against *E. coli* ATCC 25922. All the assayed extracts were cytotoxic to human neuroblastoma SH-SY5Y cells with a dose-dependent effect. Additionally, skin extracts were more potent in inducing antiproliferative effects than flesh extracts. Among flesh extracts, Moradita was the most potent. Finally, viability was lower or even null in both bacteria and human cells treated with commercial 5-CGA. Moradita flesh appears as a desirable source for important health-promoting substances, since it exerted both bactericidal and cytotoxic activities. This study enlarges the knowledge on bioactivity of potato polyphenols; however, further work is needed to elucidate the specific active compound/s.

## Figures and Tables

**Table 1:** Phenolic acid and anthocyanidin content in skin and flesh of tubers of the four selected potato cultivars.

Compound	Cultivar																			
	Moradita					Waicha					Santa María					Summerside				
	Skin		Flesh		*R*	Skin		Flesh		*R*	Skin		Flesh		*R*	Skin		Flesh		*R*
5-CGA	970.85 ± 51.00	(69.3)	63.15 ± 6.65	(94.2)	15.4	184.10 ± 23.16	(26.3)	55.70 ± 11.90	(18.3)	3.3	1145.25 ± 46.60	(72.8)	877.30 ± 47.15	(87.0)	1.3	304.55 ± 49.95	(78.9)	26.30 ± 3.35	(55.2)	11.6
4-CGA	212.25 ± 9.65	(15.1)	ND	—	—	261.45 ± 1.55	(37.3)	153.75 ± 2.40	(50.6)	1.7	248.30 ± 65.35	(15.8)	101.05 ± 15.60	(10.0)	2.5	42.55 ± 8.00	(11.0)	13.10 ± 0.70	(27.5)	3.2
3-CGA	97.25 ± 0.45	(6.9)	ND	—	—	201.55 ± 1.55	(28.8)	91.65 ± 1.65	(30.1)	2.2	134.70 ± 36.35	(8.6)	23.50 ± 8.10	(2.3)	5.7	22.10 ± 6.75	(5.7)	6.85 ± 0.20	(14.4)	3.2
CA	91.35 ± 6.70	(6.5)	0.85 ± 0.15	(1.3)	107.5	39.05 ± 13.10	(5.6)	3.10 ± 1.70	(1.0)	12.6	32.55 ± 4.65	(2.1)	4.45 ± 2.70	(0.4)	7.3	15.95 ± 1.70	(4.1)	0.50 ± 0.30	(1.0)	31.9
FA	30.30 ± 6.80	(2.2)	3.00 ± 0.40	(4.5)	10.1	14.20 ± 3.80	(2.0)	ND	—	—	11.50 ± 3.95	(0.7)	2.55 ± 0.40	(0.3)	4.5	1.25 ± 0.35	(0.3)	0.90 ± 0.25	(1.9)	1.4
Total	1402.05 ± 74.70	(74.4)	67.00 ± 7.35	(100)	21.3	700.35 ± 43.15	(67.2)	304.20 ± 17.65	(100)	2.3	1572.30 ± 156.90	(63.0)	1008.85 ± 73.95	(61.3)	1.6	386.40 ± 66.75	(100)	47.65 ± 4.80	(100)	8.1
phenolic acids																				

Cyanidin	1.50 ± 0.65	(0.3)	ND	—	—	5.85 ± 1.65	(1.7)	ND	—	—	1.25 ± 0.40	(0.1)	5.10 ± 2.40	(0.8)	0.2	ND	—	ND	—	—
Petunidin	440.65 ± 10.30	(91.2)	ND	—	—	ND	—	ND	—	—	ND	—	ND	—	—	ND	—	ND	—	—
Pelargonidin	ND	—	ND	—	—	267.55 ± 1.15	(78.1)	ND	—	—	781.95 ± 49.15	(84.7)	494.20 ± 50.65	(77.5)	1.6	ND	—	ND	—	—
Peonidin	21.10 ± 2.60	(4.4)	ND	—	—	51.30 ± 2.50	(15.0)	ND	—	—	127.00 ± 13.35	(13.7)	126.40 ± 47.00	(19.8)	1.0	ND	—	ND	—	—
Malvidin	20.10 ± 7.80	(4.1)	ND	—	—	17.90 ± 2.25	(5.2)	ND	—	—	13.80 ± 8.25	(1.5)	12.30 ± 2.05	(1.9)	1.1	ND	—	ND	—	—
Total	483.35 ± 21.25	(25.6)	0	—	—	342.55 ± 3.60	(32.8)	0	—	—	923.95 ± 71.10	(37.0)	638.05 ± 102.15	(38.7)	1.4	0	—	0	—	—
anthocyanidins																				

*Note*. Metabolite levels were determined by HPLC-DAD and expressed as *µ*g/g DW ± SD from at least three independent extractions. Numbers in parentheses indicate the % of each compound(s) with respect to the corresponding total. *R*: skin-to-flesh ratio; ND: not detected.

**Table 2:** MBC values (µg/mL) in *E. coli* ATCC 25922 or *E. coli* O157 subjected to different treatments for 24 h.

*E. coli*	MBC (µg/mL)										
	Extraction solvent	Gentamicin	Commercial 5-CGA	Moradita		Waicha		Santa María		Summerside	
				Skin	Flesh	Skin	Flesh	Skin	Flesh	Skin	Flesh
ATCC 25922	—	1	512	—	1024	—	—	—	—	1024	—
O157	—	2	2048	—	—	—	—	—	—	—	—

*Note*. —, no inhibition effect detected. Data are representative of at least two independent experiments.

## Abbreviations

CA: Caffeic acid
5-CGA: Chlorogenic acid
4-CGA: Cryptochlorogenic acid
3-CGA: Neochlorogenic acid
DPPH: 2, 2-diphenyl-1-picrylhydrazyl
FA: Ferulic acid
HPLC-DAD: High-performance liquid chromatography with diode-array detection
MBC: Minimum bactericidal concentration.

## Supplementary Materials

Supplementary Materials Supplementary Figure S1: HPLC-DAD chromatograms of extracts from potato tuber skin and flesh. Shown are representative profiles at 320 nm for CGA isomers and phenolic acids and at 510 nm for individual anthocyanidins when present. Cy: cyanidin; Pt: petunidin; Pg: pelargonidin; Pn: peonidin; Mv: malvidin.
